# When Healthcare Professionals Disagree: Finding the Right Balance

**DOI:** 10.21315/mjms2024.31.4.19

**Published:** 2024-08-27

**Authors:** Yusrita Zolkefli

**Affiliations:** 1 PAPRSB Institute of Health Sciences, Universiti Brunei Darussalam, Brunei Darussalam; 2 School of Health in Social Science, The University of Edinburgh, Edinburgh, United Kingdom

**Keywords:** ethics, healthcare professionals, conflict, doctors, nurses

## Abstract

Healthcare disagreements are common, but recognising the causes is essential to reaching a moral consensus. The article describes the challenges associated with resolving the disagreements. Therefore, a systematic and timely team-based discussion, ethics consultation with ethicists and the hospital ethics committee, active participation of all members’ discussions, and scheduled debriefings are pragmatic ways to find balance when healthcare professionals disagree. Teams need these attempts to build consensus and make moral decisions. It also fosters harmony, prioritises patient and team interests and, most importantly, keeps the team intact.

## Introduction

In general, the provision of patient care is perceived and considered differently by various groups of healthcare professionals. These differences become apparent when carrying out routine tasks, such as coordinating the discharge of patients from the hospital. There is a possibility that these decisions will directly oppose the desires and priorities communicated by patients, their families and healthcare professionals. The intricate and nuanced nature of medical decision-making is demonstrated by the multiple facets involved. There are four primary causes of moral disagreements: i) differences in the assessment or prioritisation of harms and benefits, ii) ideological differences regarding the nature of human beings, iii) different interpretations of moral rules or principles, and iv) differences in the extent of moral obligations, specifically in terms of who is protected and who is not (1). Non-clinical factors, such as politics, culture, tradition and societal values, influence the phenomenon of moral disagreements. The progress of moral disagreements into ethical conflicts is associated with avoiding or delaying discussions, unresolved differences, inflexible moral positions and disregard or rejection of other moral perspectives (2).

### Significance of Moral Disagreements

Moral disagreements have an impact on the psychological safety of staff members. Healthcare professionals are able to undertake interpersonal risks in the interest of teamwork and patient safety due to psychological safety (3). Consequently, when psychological safety is lacking, team members are reluctant to express their viewpoints, suggestions or concerns and may even repress them (4). Moral disagreements can also cause team friction and lower treatment quality. An interview with various healthcare professionals at a Swiss teaching hospital found that 4 out of 10 conflict situations affect patient care. Interprofessional disagreements often cause long hospital stays and healthcare delays. While intraprofessional conflicts frequently arose from a lack of patient-centred care, and in some cases, team conflicts were sparked by the perception of inadequate quality of care (5). Disagreements are also possible because decisions are unconsciously accepted as routine and assumptions are unquestioned. This leads to errors in thinking or cognitive bias, which happens when intuitive or rapid thinking is utilised to conclude information instead of analytical or mindful thinking (6). A recent study found that cognitive biases may affect clinical judgements in advanced dementia patients. Consequently, healthcare professionals hesitate to provide potentially beneficial treatments to the patient if family members decline due to administrative concerns about resource allocation (7). The treatment approach has also led to passive thinking, causing healthcare professionals to consider only accepted or default care alternatives.

Meanwhile, the emotions of healthcare professionals can significantly impact the clinical decision-making process. Approximately one-third of the physicians admitted that their clinical judgement had been affected by their annoyance and empathy in response to the patient’s request (8). Another study suggested that their emotions and intuition occasionally influenced clinicians’ discharge decisions (9). As a result, the decision was inadequately examined and may lead to misleading assessments and judgements.

Additionally, ethical sensitivity is deemed a necessary component of ethical practises, whereby a high degree of sensitivity is required to recognise the moral dimension in patient care and make the correct decision (10). It has been suggested that professionals with insufficient sensitivity may fail to recognise existing moral situations and, as a result, fail to take appropriate action (11). In other words, disagreements may arise when professionals perceive ethical issues differently or when their decision-making is hindered by a lack of moral courage, resulting in diminished sensitivity (12).

### Challenges in Reconciling Disagreements

Given the frantic nature of clinical settings, an impromptu decision can often be made without adequate reflection on the ethical dimension of patient care. This generates doubts among the team members, while to some healthcare professionals, accepting the team decision is more pragmatic than resisting it, as it is the best way to demonstrate team loyalty and avoid straining professional relationships. In some situations, healthcare professionals may also ‘go with the flow’ with an already established decision. This phenomenon is known as the status quo effect, in which decisions to maintain the status quo are less likely to be regretted than decisions to change. Furthermore, reconciling moral differences is not always simple (13), particularly when it requires a degree of courage and readiness to express disagreement. It is possible for nurses, for example, to feel doubtful about their professional authority, competence and abilities, making them nervous to speak up (14).

Meanwhile, there is an increase in emotional intensity when a team has conflicting emotions and points of view (15). Confrontation can be misinterpreted as aggression in some cultural contexts, suggesting that all forms of disagreement are viewed negatively. This results in an ‘epistemic silence’ or deliberate avoidance of confrontation (16). The institutional structure of hospitals, which does not actively promote or adequately support interprofessional collaboration, also hinders the efficacy of interprofessional communication and collaboration (17).

Also challenging is the prioritising policy, such as clinicians and managers who hold different views regarding resource allocation prioritisation (18). Conflicting team goals make this harder. For example, nurses believe their role is to provide empathy and facilitate dignified dying, even if the medical decision is to continue intensive life-sustaining care or explore additional curative therapies (19). The challenge is further complicated by differences in professional cultures, critical appraisal of evidence and mindset. Doctors’ moral duty, for instance, is to develop clinical and scientific methods for cure. In contrast, nurses’ moral work considers the patient and family’s ‘lived experience’ in delivering medical, emotional and spiritual care (20). This causes team members to have different role expectations of each other.

Conversely, the medical system’s interests also exert an impact. A study conducted in Germany suggested that physicians were increasingly burdened with the need to prioritise the economic interests of the hospital when making patient care decisions. This pressure resulted in inappropriate care, overtreatment and undertreatment (21). Furthermore, the hospital’s emphasis on its reputation may influence such conflicts of interest. As an illustration, certain healthcare facilities in the United States employ prolonged hospitalisation and the continuation of treatments that sustain patient life for over 30 days after surgery without considering the patient’s voluntary consent to unnecessary life-prolonging treatment in pursuit of high-quality indicators (22).

### The Way Forward

To keep the team intact, healthcare professionals need to remain respectful and diplomatic when addressing moral disagreements within the team to reach a consensus. To achieve this, a systematic and timely team-based discussion is essential to achieving this goal. At first, the healthcare team’s viewpoint on stressors in each clinical unit and the work itself would facilitate the development of educational interventions and training courses that are carefully tailored to the needs of all staff members. This is followed by the adoption of frameworks such as The Proactive Clinical Ethics Framework, which enables a timely team-based ethics dialogue to resolve moral disagreements (2). In addition, the interprofessional rounding approach, for example, promotes the teaching of difficult conversation skills, ethicist-led discussion, and the clarification of decision-making (23).

Next, all staff members should actively participate in team-based discussions during multidisciplinary team sessions or meetings. While most healthcare professionals attempt to create a cohesive team environment, disagreements can improve patient care. Thus, they should contribute to the discussion. A crucial responsibility in this context is that of the team leader, who ensures that every member is actively involved in the decision-making process and has the opportunity to express their expert viewpoints. Active participation promotes team cohesion by facilitating the exchange of skills, knowledge and experience among team members, all for the mutual benefit of the patient and the team. Healthcare providers may also need to consult other professionals for additional perspectives. It improves discussions and clarifies moral dilemmas in ethics consultation (19). Ethicists, including those on hospital ethics committees, support positive discourse and open discussion to comprehend difficult topics better. Finally, there are scheduled debriefings after major incidents. Such meetings allow a healthcare team to review the clinical interaction, evaluate individual and team performance, identify errors, and develop reflective learning strategies (24). Implementing this approach will make the team more receptive to and cognisant of early signs of moral disagreements.

## Conclusion

Recognising that healthcare professionals will disagree on some moral issues is vital, especially when the care is complex and they have varied information, duties, training, values and beliefs. In finding the balance when healthcare professionals disagree, several pragmatic strategies are offered: timely team-based discussion, ethics consultation with ethicists and the hospital ethics committee, active participation in team-based discussions and scheduled debriefings after major incidents. These combined efforts are needed to reach team consensus and morally sound decisions. It also helps to maintain harmonious relationships, prioritise the patient’s and team’s interests, and, most importantly, keep the team intact.
